# Good News and Bad News About Incentives to Violate the Health Insurance Portability and Accountability Act (HIPAA): Scenario-Based Questionnaire Study

**DOI:** 10.2196/15880

**Published:** 2020-07-20

**Authors:** Joana Gaia, Xunyi Wang, Chul Woo Yoo, G Lawrence Sanders

**Affiliations:** 1 State University of New York at Buffalo Buffalo, NY United States; 2 Hankamer School of Business Baylor University Waco, TX United States; 3 Florida Atlantic University Boca Raton, FL United States

**Keywords:** cyber security, data security, Health Insurance Portability and Accountability Act, motivation, economics of crime, rational choice theory

## Abstract

**Background:**

The health care industry has more insider breaches than any other industry. Soon-to-be graduates are the trusted insiders of tomorrow, and their knowledge can be used to compromise organizational security systems.

**Objective:**

The objective of this paper was to identify the role that monetary incentives play in violating the Health Insurance Portability and Accountability Act’s (HIPAA) regulations and privacy laws by the next generation of employees. The research model was developed using the economics of crime literature and rational choice theory. The primary research question was whether higher perceptions of being apprehended for violating HIPAA regulations were related to higher requirements for monetary incentives.

**Methods:**

Five scenarios were developed to determine if monetary incentives could be used to influence subjects to illegally obtain health care information and to release that information to individuals and media outlets. The subjects were also asked about the probability of getting caught for violating HIPAA laws. Correlation analysis was used to determine whether higher perceptions of being apprehended for violating HIPAA regulations were related to higher requirements for monetary incentives.

**Results:**

Many of the subjects believed there was a high probability of being caught. Nevertheless, many of them could be incentivized to violate HIPAA laws. In the nursing scenario, 45.9% (240/523) of the participants indicated that there is a price, ranging from US $1000 to over US $10 million, that is acceptable for violating HIPAA laws. In the doctors’ scenario, 35.4% (185/523) of the participants indicated that there is a price, ranging from US $1000 to over US $10 million, for violating HIPAA laws. In the insurance agent scenario, 45.1% (236/523) of the participants indicated that there is a price, ranging from US $1000 to over US $10 million, for violating HIPAA laws. When a personal context is involved, the percentages substantially increase. In the scenario where an experimental treatment for the subject’s mother is needed, which is not covered by insurance, 78.4% (410/523) of the participants would accept US $100,000 from a media outlet for the medical records of a politician. In the scenario where US $50,000 is needed to obtain medical records about a famous reality star to help a friend in need of emergency medical transportation, 64.6% (338/523) of the participants would accept the money.

**Conclusions:**

A key finding of this study is that individuals perceiving a high probability of being caught are less likely to release private information. However, when the personal context involves a friend or family member, such as a mother, they will probably succumb to the incentive, regardless of the probability of being caught. The key to reducing noncompliance will be to implement organizational procedures and constantly monitor and develop educational and training programs to encourage HIPAA compliance.

## Introduction

### Background

The Health Insurance Portability and Accountability Act (HIPAA) of 1996 introduced legislation for protecting the privacy of personal health information. Although the health care industry in the United States is one of the most regulated industries, compliance with the regulations is variable. In 2017, more than 14.6 million people were affected by data breaches [1]. Cybersecurity reports illustrate that health care data breaches will continue to increase [1-4]. Some of these breaches are simply external malicious attacks, but they are often the result of rent-seeking and illegal behaviors of insiders [5-7]. Verizon’s 2018 Data Breach Investigations Report paints a bleak picture of the health care industry in which errors and misuse of data are widespread [8,9]. Health care is the only vertical industry that has more insiders behind breaches: 58% when compared with external actors at 42%. This is probably the reason why the majority of the US population does not trust organizations that share health care information [10-12].

The objective of this study was to identify the role that monetary incentives play in the next generation of employees when it comes to violating HIPAA regulations and privacy laws. These individuals are of particular interest because many will also become trusted insiders, with the knowledge and insight to significantly compromise organizational security systems. The research model was developed using the economics of crime and rational choice theory frameworks to identify situations where employees might engage in illegal breach behavior. Scenarios were developed for 5 situations to determine whether monetary incentives could be used to influence subjects to obtain health care information and to release that information. Approximately 35.4% (185/523) to 45.9% (240/523) of the survey participants indicated that there is a price, ranging from US $1000 to over US $10 million, that is acceptable for violating HIPAA laws. In addition, subjects were also asked about their perceived probability of getting caught for violating HIPAA laws. More than 50.1% (262/523) of the participants indicated that the probability of getting caught was more than 74.9% (392/523). Nevertheless, many of them could still be incentivized to violate HIPAA laws. The correlations between the probability of being apprehended and the level of the monetary incentive required for violating HIPAA ranged from 0.14 to 0.43.

### Related Work

#### Foundation Research on the Economics of Crime

Gary Becker’s seminal paper on the market for criminal activity posits that potential criminals examine returns on criminal activity as a function of the probability of getting caught or apprehended and the severity of the punishment [13]. He argued that criminals commit crimes when they perceive the expected benefits from crime would exceed the expected cost of crime. Becker received a Nobel Prize for his research on the economics of crime. Becker’s [14] economics of crime model has received more than 1000 citations a year, although it was published in 1968.

General deterrence theory in the information systems area is used to explore the effects of countermeasures and security policies on protecting information and improving security [15,16]. Early papers by Gopal and Sanders [17,18] examined the role of preventive and deterrent controls on software piracy. Herath and Rao [19] found that the perception of certainty of detections is related to intentions to comply with security policies, but that severity of penalty did not have a deterrent effect. However, deterrence theory research results have been inconsistent and contradictory, and more attention is needed on the theoretical and methodological foundations [20].

General deterrence theory is based on Gary Becker’s theory that criminal behavior is deterred when the expected loss (penalty of violating the law) is greater than the expected gain. Many studies involving deterrence theory have focused primarily on the effect of penalties [21]. A framework known as routine activity theory states that a crime can arise from changes in the structured situation or environmental setting, and 4 elements—value, inertia, visibility, and access—would affect the suitability of a target of crime [16,22]. The following paragraphs provide details on the conceptual foundations of the Becker model.

Engaging in criminal activity involves a choice with consequences and opportunities, where individuals perceive them differently. They can be deterred if there is a likelihood of punishment, and the punishment is severe [23]. The market model for crime assumes that offenders, victims, and law enforcement engage in optimizing behavior related to their preferences and that offenders have expectations about returns, the propensity for being caught, and the resulting punishment [23]. This model assumes that potential participants in illegal activities are rational economic actors. Empirical research in the area typically uses an event study that examines whether changes in laws, punishment (incarceration and fines), increases in law enforcement, drug usage, and the economy lead to increases or decreases in criminal activity [24-26].

**Figure 1:**
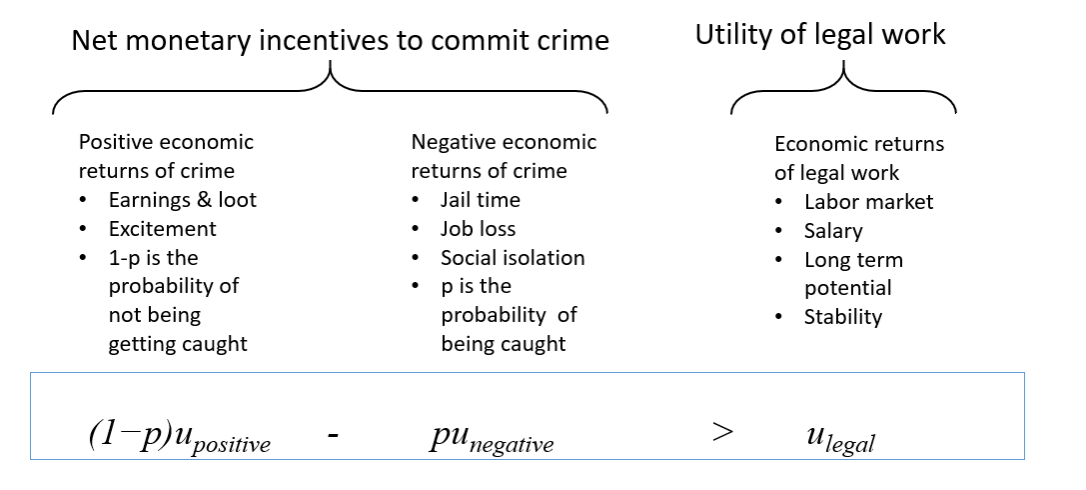
The Becker crime utility model.

Wrongdoers use a calculus of rational choice to determine whether to engage in criminal activity [13,27]. An individual will commit a crime if the inequality in Figure 1 holds [28].

The *u_positive_* term is the expected utility obtained by the potential perpetrator if he or she commits the crime. This utility can mean both monetary and nonmonetary gains. The *u_negative_* term is the expected utility resulting from being apprehended and the ensuing punishment. The *p* term is the probability of being apprehended or getting caught. This is a perception of the risk of offending [27]. The *u_legal_* term is the utility derived when he or she does not commit the crime. If the net expected gains from the left side of the inequality are greater than the utility of engaging in legal work on the right side, then the individual will commit the crime.

We illustrate a simplified model of the calculations using 2 equations that form the basis of the model. The criminal will weigh the costs and benefits in the following way:

Benefits=Probability of success × (Gains from crime + Other benefits)

Costs=Probability of getting caught × (Punishment for getting caught + Other costs)

Assume that the expected profits to the potential perpetrator for engaging in illegal activity is US $10,000 and that the probability of success or not getting caught is 90%. The other benefits may be that the potential perpetrator finds excitement from participating and even camaraderie. The utility of these other benefits can be translated into US $2000. Therefore, the total potential benefit is US $10,800 (0.90 × [US $10,000 + US $2000]).

On the costs side, let us assume that the perpetrator perceives that fines of US $16,000 are typically levied as punishment for this type of crime. The other costs might be a loss of job for a few months and social isolation that can be translated into US $6000. The probability of getting caught is 0.10. The total potential cost for engaging in this activity if caught is US $2200 (0.10 × [US $16,000 + US $6,000]).

As the benefits (US $10,800) exceed the costs (US $2200), the individual might engage in criminal activity if this amount of money is perceived as sufficient. As the results of this study show, sometimes there are never enough benefits for people to engage in illegal activities. The other costs are sometimes perceived as being too large, and this translates to a high level of disutility. The other costs could include the loss of a job, prison time, and social desirability effect from a large social network.

There are ongoing discussions and controversy about utility theory and the use of rational decision making among traditional and behavioral economists. Behavioral economists do not abandon the notion that humans can be rational, but they think that there are situations where decision making is less than rational and that more robust models are needed to understand the vagaries of human behavior [29-33]. Our research draws on a combination of traditional economics and behavioral economics to understand the role of incentives in modeling choice behavior related to criminal activity. Empirical evidence supports the role of incentives in terms of labor market experiences and perceptions of the probability of being apprehended and incarcerated [34].

The economics of crime model posits that deterrence will work to counter monetary gains if the penalties are large and if there is a certain level of risk of being caught. There is some empirical evidence that the criminal justice system’s ability to deter crime is weaker than thought [26]. However, vibrant labor markets and high manufacturing wages appear to be very effective in deterring crime. In a recent review on the economics of crime, Stephen Levitt of Freakonomics fame [35,36] predicts that there will be fewer research studies on the economics of crime because of declining criminal activity:

In some sense, however, public policies to reduce crime (many of them informed by economic thinking) have proven too successful from the perspective of the academic interested in studying crime. With the crime rate at less than half the level it was two decades ago in the United States and lower almost everywhere else in the world as well, the demand for crime research has no doubt also been diminished[37]

Although it may be true that certain crimes are decreasing, criminal activity involving cybercrime, information security breaches, and privacy intrusions have resulted in substantial dollar losses. HIPAA noncompliance has become a very serious problem. As noted earlier, in 2017, more than 14.6 million people were affected by data breaches, and in the health care industry, errors and misuse of data are widespread [1].

We agree, in part, with Levitt’s assertion that academic research has made some gains; however, we believe that the research is at an early stage when it comes to cybercriminal activity, particularly in health care practice. There is evidence that the number of security incidents has decreased, but the dollar amount of financial losses per incident has increased [37]. Underreporting of cybercrime is an elephant-in-the-room problem. Companies are sometimes reticent to report cybercrime because they are embarrassed, and they fear that they will lose customers.

#### Insider Attacks

Insiders can be current and former employees, contractors, and business partners that have access to an organization’s network, system, or data. Insiders can engage in malicious or unintentional activities that negatively affect the confidentiality, integrity, and availability of an organization’s information system [38,39].

A recent large-scale, country-wide study found that cyberattacks by outsiders are strategic and often motivated by economic incentives [40]. These attacks can adversely affect business operations and compromise sensitive customer information. However, it appears that trusted insider threats, traced to existing employees, are also related to economic incentives.

The focus of this research is on insider attacks because they account for a substantial portion of privacy violations, including funds embezzlement; pilfering of trade secrets; theft of customer information and competitive information; and a variety of illegal, fraudulent activities [41], and they can also result in significant losses [42]. Malicious insiders can cause more damage to the organization than traditional hackers [43]. The average cost of an insider attack is US $8 million per year [44], but the fallout from a breach can lead to long-term loss of customers, lawsuits, and damaged reputations.

In some instances, insider security breaches occur because of negligence. For example, some people do not know that they are not supposed to maintain social security numbers in a temporary file or email a medical diagnosis to another doctor without obtaining permission. Insiders pose a considerable threat to organizations as they can bypass several security measures using their knowledge and access to the systems [45]. The motives behind malicious attacks are diverse, including seeking revenge and retribution, thrills, anarchy, and curiosity. Financial motives, however, are the undercurrent of most attacks and include reasons such as student loan debt, financial pressures caused by health care needs or mounting personal debt (eg, credit cards and gambling), or loss of financial stability (job loss or demotion). Threats from trusted insiders are difficult to detect, are embarrassing, damage the reputation of the organization, are often destructive, and cause serious operational disruptions [46].

### Hypotheses Development

**Figure 2:**
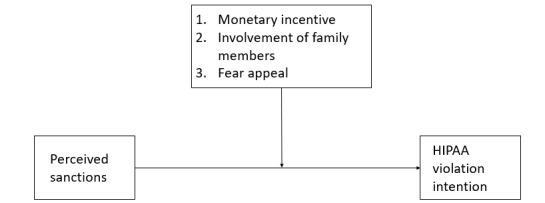
The conceptual model. HIPAA: Health Insurance Portability and Accountability Act.

The primary objective of this study was to identify the role that monetary incentives play in violating HIPAA regulations and privacy laws in the next generation of employees. The conceptual model is presented in Figure 2. The research hypotheses draws on the economics of crime and rational choice theory frameworks to identify situations where employees might engage in illegal breach behavior.

Our first research hypothesis examines the role of the level of monetary inducements and the perceived probability of being apprehended in violating HIPAA laws.

Hypothesis 1: Higher perceptions of being apprehended for violating Health Insurance Portability and Accountability Act regulations are related to higher requirements for monetary incentives.

Our second research hypothesis focuses on the role of the situational or personal context in violating HIPAA laws. Under the specific context in which a family member or friend needs critical medical assistance that is not covered by insurance, we believe that the relationship will not be as strong as the relationship in Hypothesis 1. Sometimes, there are compelling personal reasons for committing offenses [41]. They can include medical bills, credit card debt, addictions, and the desire to help a family or friend in need. Scenario 4 involves the need to pay for an experimental operation for the subject’s mother. Scenario 5 involves the need to pay for an ambulance airlift for a close friend.

Hypothesis 2: Higher Perceptions of Being Apprehended for Violating Health Insurance Portability and Accountability Act Regulations are Related to Higher Requirements for Monetary Incentives When the Personal Context Involves a Family Member or Friend, and the Strength of the Relationship is Not as Strong as in Hypothesis 1.

The last objective of this study was to determine if the perceived risk or probability of getting caught could be modified by using fear appeals as a deterrent [20]. Approximately 50% of the subjects were targeted to receive information related to real people receiving fines and jail time for violating HIPAA laws (Multimedia Appendix 1). This information is a *fear treatment*, and it is used as a deterrent in this study [47,48].

Hypothesis 3: The group receiving the fear appeal treatment will have higher perceptions of the probability of being caught violating HIPAA regulations than the group who did not receive the fear appeals treatment.

## Methods

### Participants

The local institutional review board approved the protocol for the pilot study and the main study. A questionnaire was developed to examine the relationships among an individual’s propensity to reveal private health care information when offered a monetary incentive and the subject’s perception of getting caught violating HIPAA laws. The pilot study involved medical residents and individuals in an executive MBA program, some of who work in the health care industry as executives. After collecting data for the pilot study, significant time was spent in refining the instrument and scenarios to avoid the complexity involved in estimating probabilities and trade-offs found in many research studies involving scenarios and simulated games used to evaluate choice behavior. The data were collected in May 2018.

An important consideration in designing the survey was obtaining information from the subjects on the probability of getting caught if they violated health care regulations. As noted earlier, the questionnaire items were anchored using numerical probabilities and verbal labels because this approach has proven to be a very effective method for eliciting probabilities [49], and it counters some of the measurement problems encountered in measuring perceived arrest rates involved in studies of rational choice theory [50].

The questionnaire was refined and distributed to 574 students in an undergraduate information technology course. This was a voluntary survey, and credits were given for completing the questionnaire. We chose an undergraduate sample because they were more computer proficient, they will be entering the workforce in the immediate future, they are not as aware of HIPAA compliance regulations, and they are less concerned with social desirability issues. These students have majored in business IT, and they have largely been trained for business evaluation and business decision making, but not much on health care, especially the regulations or laws in health care. This is a closed survey that was only open to this particular sample, and we used a password to ensure this.

In social science research, social desirability bias is a type of response bias that is the tendency of survey respondents to answer questions in a manner that will be viewed favorably by others. It can take the form of overreporting *good behavior* or underreporting *bad* or undesirable behavior [51]. Social desirability bias occurs when subjects are less prone to answer questions truthfully, which could diminish their social prestige [52]. We assert that the medical interns and the executive MBA participants in the pilot test were deeply concerned with social desirability issues as well as the potential loss of high incomes. That is why we did not revisit that population in the main study. Individuals with high status tend to overreport *good behavior* and underreport *bad behavior*. Social desirability bias is a problem in studies involving abilities, personality, and illegal activities. Subjects with high incomes and status tend to deny illegal acts. In the pilot study, only 6% (6/96) of the participants (3 of the medical residents and 3 of the executive MBAs) succumbed to incentives to violate HIPAA laws. The amount of money required by these individuals ranged from US $50,000 to US $1 billion.

Students in the main study group were given 3 extra points in their final exam for participating in the anonymous survey regardless of completion. We removed subjects with more than 10% (1/10) missing values and subjects who took less than 3 min to complete the survey. The final data set consisted of 523 subjects out of the initial 574 survey participants.

The study subjects consisted of 60% males and 40% females, and their average age was 21 years. The study population consisted of 45% whites, 4% blacks, 4% Hispanics, 45% Asians, and 3% others.

### Overview of the Scenarios

Scenarios were adapted from an earlier HIPAA compliance study [53] and redeveloped for 5 situations to determine if monetary incentives could influence subjects to obtain health care information and to release that information to individuals and media outlets (Textbox 1). Multimedia Appendix 2 also illustrates an example of the survey question that elicited a response on how much money a subject would accept to reveal information and their perception of the probability of being apprehended for the nursing scenario. The first 3 scenarios do not incorporate a personal or family situation involved in deciding to accept a monetary incentive. We refer to these scenarios as having no personal context. In addition, we use different income levels in the scenarios, resulting in a differential influence of monetary incentives on insiders’ deviant behavior decisions. The salaries in the first 3 scenarios were US $30,000, US $200,000, and US $60,000. We included these salary levels to examine income sensitivities.

Scenarios 4 and 5, however, involve personal or situational contexts. There are situational and transitory influences that lead to committing offenses [41], and they can include financial problems, medical bills, and addictions. Scenario 4 involves the need to pay for an experimental operation for the subject’s mother. Scenario 5 involves the need to pay for an ambulance airlift for a close friend. The 2 scenarios incorporate the concept of the value of life and the notion of *identified lives* as distinct from *statistical lives* [30,54]. The economics literature on the value of life distinguishes between identified and statistical lives. Statistical lives have no face or a personal context. Examples include the number of cancer cases that have occurred, the number of lives lost in traffic accidents, and lives lost in natural disasters, whereas identified lives have faces, names, family, and a historical legacy. Identified lives have a personal context.

**Overview of the scenarios.**

**Scenario 1: Nurse’s aide, no personal context**

Suppose you are a nurse’s aide at a hospital and you earn US $30,000 per year. A friend asks you to get them some information on a patient you have been caring for. What amount of money would you receive to make this acceptable?

**Scenario 2: Doctor, no personal context**

Suppose you are a doctor at a hospital and you earn US $200,000 per year. A very close friend asks you to access patient information to help them in an upcoming legal battle. What amount of money would you receive to make this acceptable?

**Scenario 3: Insurance local celebrity, no personal context**

Suppose you work for an insurance company and make US $60,000 per year. A relative asks you to get insurance data on a famous local celebrity from the organization you work for. What amount of money would you receive to make this acceptable?

**Scenario 4: Your mother needs an experimental treatment, personal context**

Your mother has just been diagnosed with a rare condition that causes kidney failure and is fatal if untreated. This condition can be treated, but the treatment is still considered experimental and is therefore not covered by health insurance, nor is it eligible for any type of financial assistance. The treatment is available both nationally and internationally and costs US $100,000. A media outlet approaches you to get information about a famous politician and offers to pay you US $100,000 for that information. This money can save your mother’s life. Would you accept the payment from the media outlet and give the money to your mother?

**Scenario 5: Best friend needs air medical transportation, personal context**

Your best friend has been in an all-terrain vehicle accident in a rural area of Kansas. He or she has life-threatening injuries and needs air medical transportation to receive lifesaving medical care. The medical air evacuation is not covered by insurance and costs US $50,000. Your best friend will not survive ground transportation or local medical care. A media outlet offers you US $50,000 to obtain the health care records of a famous reality television star. This money can save your best friend’s life. Would you accept the payment from the news outlet to give the money to your best friend?

**Each scenario also included the following question:**

What do you think is the likelihood of getting caught if you accept the money?

Extremely unlikely (0%)

Moderately unlikely (7%)

Slightly unlikely (25%)

Neither likely nor unlikely (50%)

Slightly likely (75%)

Moderately likely (93%)

Extremely likely (100%)

### Deterrent Treatment

As noted earlier, we also included a deterrent treatment in the study for half of the participants in the study [19]. The treatment consisted of short vignettes that described instances where individuals received fines and were sentenced for violating HIPAA regulations (Multimedia Appendix 1). Half of the subjects received the treatment.

### Research Design

In this section, we provide an overview of the study design. First, 574 students in an undergraduate information technology course voluntarily participated in this survey. Credits were given for completing the questionnaire. Second, half of the participants were given the deterrent treatment, which consisted of short vignettes that described the possible punishments for violating HIPPA regulations. Third, all participants, including both the treated and nontreated ones, completed the survey, where the 5 scenarios were presented. The average completion time was 8.5 min. Therefore, given the clear logic of the survey and the time needed to complete the survey, we believe that survey fatigue is not a serious concern in our study.

## Results

### Main Findings

We used correlation analysis to explore the relationship between the net monetary incentive to commit a crime and the perceived probability of being apprehended in Hypothesis 1. Hypothesis 1 was supported. It shows that higher perceptions of being apprehended for violating HIPAA regulations are related to higher requirements for monetary incentives. The correlations between the probability of getting caught and the amount of money that the subjects would accept to provide the information were 0.44 (*P*<.001) for the nursing scenario, 0.25 (*P*<.001) for the doctor scenario, and 0.43 (*P*<.001) for the insurance scenario. Differences in income can explain the differences in the correlations for the nurse/insurance scenarios as compared with the doctor scenario. The nurse aide’s salary was US $30,000; the doctor’s salary was US $200,000; and the insurance agent’ s salary was US $60,000. Referring back to the Becker crime utility model in Figure 1, the monetary incentives to commit a crime on the left side would have to be substantially greater than the utility of legal work on the right side. We had posited that the students would not be aware of HIPPA laws; however, approximately 51% agreed or strongly agreed that they were aware of HIPPA regulations. This variable, however, did not have a statistically significant effect on the results when included in the analysis.

These results provide strong support for Hypothesis 1, showing that higher perceptions of being caught for violating HIPAA regulations are related to higher requirements for monetary incentives. Individuals in the study that perceive higher levels of risk of being caught, in essence, will require more money to participate in an illegal act.

**Figure 3:**
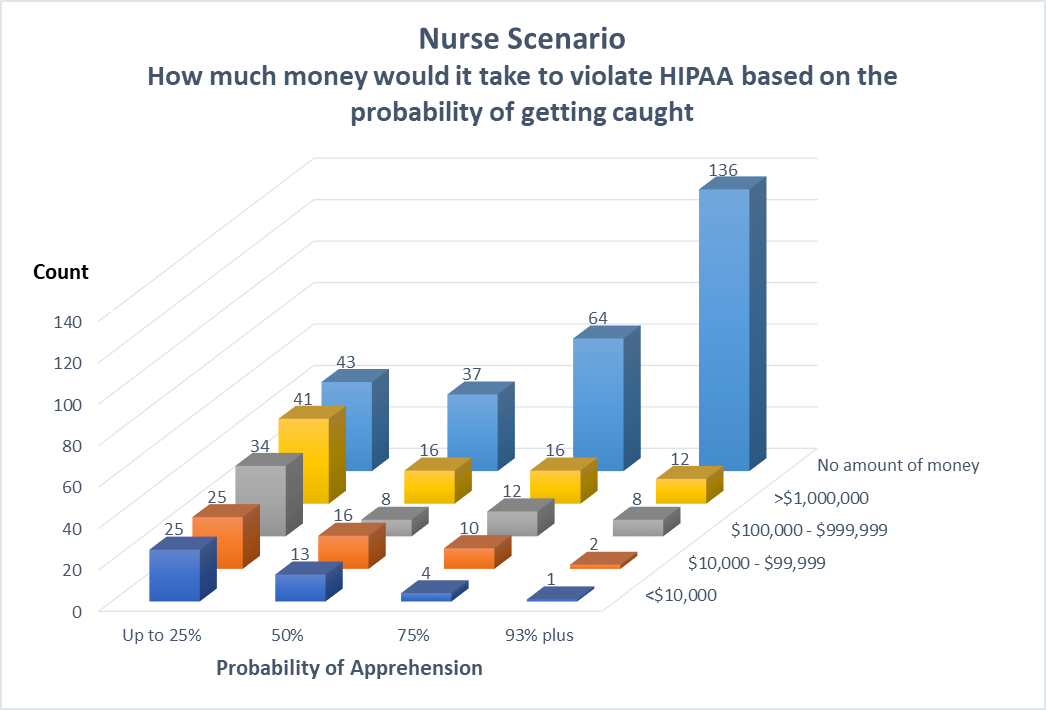
Nursing scenario results.

To improve the readability of the instrument crosstabs, we collapsed the amount of money from 11 to 5 categories and the probability of getting caught from 7 to 3 categories. Many of the subjects felt that the probability of getting caught for violating a HIPAA law was very high, greater than 93%. In the nursing scenario, 30% (157/523) of the participants thought the probability of getting caught was greater than 93%, and in the doctor scenario, 50% (261/523) of the participants thought the probability of getting caught was greater than 93%. In the insurance scenario, 39% (204/523) of the participants thought the probability of getting caught was greater than 93%. In the mother scenario, it was 37% (194/523), and in the best friend scenario, it was 38% (199/523). Although many of the individuals in the study believed there was a high probability of being caught, a good number of them could be incentivized to violate HIPAA laws. Tables 1-5 show the results. Figure 3 reflects the general trend of the relationship regarding the amount of money it would take to violate a HIPAA regulation based on the probability of getting caught.

**Table 1:** Nurse, no personal context (scenario 1).

Scenario 1		Perceived probability of getting caught (R=0.438; *P*<.001; 95% CI 0.36-0.52)									
		≥25%		50%		75%		≤93%		Total, n (%)	
**Amount of money willing to receive (US $), n**											
	<10,000		25		13		4		1		43 (8)
	10,000-99,999		25		16		10		2		53 (10)
	100,000-999,999		34		8		12		8		62 (12)
	>1,000,000		41		16		16		12		85 (16)
No amount of money, n		43		37		64		136		280 (54)	
Total, n (%)		168 (32)		90 (17)		106 (20)		159 (30)		523 (100)	

**Table 2:** Doctor, no personal context (scenario 2).

Scenario 2		Perceived probability of getting caught (R=0.282; *P*<.001; 95% CI 0.20-0.36)				
		≥25%	50%	75%	≤93%	Total, n (%)
**Amount of money willing to receive (US $), n**						
	<10,000	7	3	5	2	17 (3)
	10,000-99,999	9	11	6	9	35 (7)
	100,000-999,999	14	9	12	8	43 (8)
	>1,000,000	33	12	16	29	90 (17)
No amount of money, n		48	23	52	215	338 (65)
Total, n (%)		111 (21)	58 (11)	91 (17)	263 (50)	523 (100)

**Table 3:** Insurance company, no personal context (scenario 3).

Scenario 3			Perceived probability of getting caught (R=0.282; *P*<.001; 95% CI 0.20-0.36)								
			≥25%		50%		75%		≤93%		Total, n (%)
**Amount of money willing to receive (US $), n**											
	<10,000	7		3		5		2		17 (3)	
	10,000-99,999	9		11		6		9		35 (7)	
	100,000-999,999	14		9		12		8		43 (8)	
	>1,000,000	33		12		16		29		90 (17)	
No amount of money, n			48		23		52		215		338 (65)
Total, n (%)			111 (21)		58 (11)		91 (17)		263 (50)		523 (100)

**Table 4:** Personal context: your mother needs an experimental treatment (scenario 4).

Scenario 4		Perceived probability of getting caught (R=0.25; *P*<.001; 95% CI 0.17-0.33)				
		≥25%	50%	75%	≤93%	Total, n (%)
**Willing to receive US $100,000, n**						
	No	8	15	22	67	112 (21)
	Yes	82	90	114	124	410 (79)
Total, n (%)		90 (17)	105 (20)	136 (26)	191 (37)	522 (100)

**Table 5:** Personal context: best friend needs air medical transportation (scenario 5).

Scenario 5		Perceived probability of getting caught (R=0.14; *P*<.001; 95% CI 0.05-0.23)				
		≥25%	50%	75%	≤93%	Total, n (%)
**Willing to receive US $50,000, n**						
	No	24	36	34	88	182 (35)
	Yes	67	75	87	109	338 (65)
Total, n (%)		91 (18)	111 (21)	121 (23)	197 (38)	520 (100)

The magnitude of the number of individuals who would receive monetary incentives was not expected. We did postulate that there would be some individuals who could be incentivized to violate HIPAA laws, but we thought it would be a small number. In the pilot study, the subjects were medical interns and students enrolled in an executive MBA program. Only 6% (6/96) of the participants (3 medical residents and 3 executive MBAs) succumbed to incentives and violated the HIPAA laws. The amount of money required by these individuals ranged from US $50,000 to US $1 billion. We realize that individuals with high-income potential (medical interns and executive MBAs) would be less prone to violating health care laws, but we did not expect such a dramatic difference.

In the main study, 47.0% (246/523) of the participants received the money in the nursing scenario, 35.0% (183/523) of the participants in the doctor scenario, and 44.9% (235/523) of the participants in the insurance scenario. Again, differences in income might explain the difference, in part. The nurse aide’s salary was US $30,000, the doctor’s salary was US $200,000, and the insurance agent was US $60,000. Referring back to the Becker crime utility model in Figure 1, the monetary incentives to commit a crime on the left side would have to be substantially greater than the utility of legal work on the right side.

Hypothesis 2 is supported. Recall that it postulates that higher perceptions of being apprehended for violating HIPAA regulations are related to higher requirements for monetary incentives when the personal context involves a family member or friend. However, the strength of this relationship is not as strong as that of the relationship in Hypothesis 1.

Point-biserial correlations are used when there is a dichotomous variable involved. The subjects could answer either a yes or no whether they would accept money to violate a HIPAA regulation. The point-biserial correlation between the probability of getting caught and whether the subjects would accept US $100,000 from a media outlet to pay for an experimental treatment was 0.25 (*P*<.001). The point-biserial correlation between the probability of getting caught and whether the subjects would accept US $50,000 from a media outlet to pay for medical evacuation was 0.14 (*P*=.001).

These correlations are not as strong as those in the first 3 scenarios. The correlations between the probability of getting caught and the amount of money that the subjects would accept to provide the information were 0.44 for the nursing scenario, 0.25 for the doctor scenario, and 0.43 for the insurance scenario.

However, there is more to the story than just the correlations. Looking at the first 3 scenarios, in which there was no personal context, we observed that 47% (246/523) of participants in the nursing scenario indicated that they would be willing to take some level of money to provide patient data, 35% (183/523) of participants in the doctor scenario indicated they would be willing to take some level of money to provide patient data, and 45% (235/523) of participants in the insurance scenario indicated they would be willing to take some level of money to provide insurance data about a celebrity. This is in stark contrast to the 2 personal context scenarios where 79% (413/523) of participants would receive money to save their mothers and 65% (340/523) of participants would receive money to save their best friends.

It is not surprising that 79% of the participants would accept money to save their mother and 65% would accept money to save their best friend. There is a strong *personal motive* to save the lives of individuals who are friends and family, even if there is a strong chance of getting caught. These results are related to how people perceive the difference between *identified lives* and *statistical lives* [30]. Statistical lives involve aggregate numbers, such as 29,000 people die from liver cancer each year. As can be expected, the concepts of statistical life and identified life are very controversial [55]. In the United States, the value of a statistical life has been identified by government agencies to be in the US $7 million range [30]. When a situation involves familiar faces and close relationships with the individual, the use of the statistical value of a life is problematic. It is very difficult to place a value on the life of a family member or close friend. Indeed, the value of a close relative may be infinite. These results support Hypothesis 2 well, which suggests that higher perceptions of being caught violating HIPAA regulations are not related to higher requirements for monetary incentives when the personal context involves a family member or friend.

Prospect theory supports the results for the personal context. Loss of a friend or family member would have a very large impact on an individual’s life. The endowment effect also comes into play [56,57]. People value things that they possess, and family and friends are important possessions that are difficult to replace. The endowment construct is related to psychological ownership, and it supports the notion that people overvalue things they perceive they own [58]. Psychological ownership occurs when an individual feels that an object is *theirs* or *mine* [59]*.* Psychological ownership usually involves some person-object relations. However, it can also be felt toward ideas, words, artistic creations, tablets, phones, people, and virtual avatars [60].

As noted earlier, the situational context matters. In the nursing example, there were 194 individuals in the study that would not receive any amount of money nor would they turn over patient information to someone. However, those same 194 individuals would take the US $100,000 to pay for an experimental procedure for their mother. The natural question is whether they would take the money because they thought that there would not be a high probability of being caught. However, 124 of the subjects indicated a high probability of getting caught (greater than 93%) but would still help their mother.

### Individuals That Are Absolutely Deterred from Violating Health Insurance Portability and Accountability Act Laws

We also counted the number of people who would not violate HIPAA laws at all. There were 14.1% (74/523) of the people in the study that would not receive any money to violate HIPAA regulations for all 5 scenarios. They are what is referred to as absolutely deterred from engaging in criminal behavior. *Absolute deterrence* occurs when individuals refrain from criminal acts because he or she perceives that any level of risk for receiving punishment and the resulting punishment is not acceptable [41,61]. In essence, the severity, certainty, and swiftness of the punishment are not acceptable to absolutely deterred individuals. It was also interesting to note that 14 people would not help their mother but would help their friend. This result is in contrast to the 85 subjects who would help their mother but would not help their friend.

### There Is No Treatment Effect

Hypothesis 3 was not supported. Recall that it postulates that the group receiving the fear treatment will have higher perceptions of being caught violating HIPAA regulations than the group who did not receive the fear treatment.

Information related to real people receiving fines and jail time for violating HIPAA laws was received by 50% of the subjects (Multimedia Appendix 1). This information is a fear treatment and is used as a deterrent [47,48]. As noted earlier, the results of studies involving treatment effects for deterrence have been inconsistent and contradictory [20]. Fear appeals use threats in the form of graphics and narrative warnings to modify behavior. The graphics and text illustrated in Multimedia Appendix 1 had little effect on the probability of getting caught. The means between the group receiving the fear appeal treatment and the group who did not receive the treatment were not statistically significant for any of the scenarios. Earlier research on software piracy and MP3 piracy found a modest, yet statistically significant, effect when the subjects were informed about punishment for software and MP3 piracy [17,62]. Sometimes, fear appeals do not work [47,63]. Possible explanations could be that (1) the degree to which an individual perceives information assets as personally relevant is highly subjective, thus potentially marginalizing the impact of the fear appeal, and (2) the conventional fear appeal rhetorical framework is inadequate in providing threat warnings when it is used in the information security context [63]. We included what would be considered as harsh sanctions as a treatment, and there was still no effect.

There is a notion of readiness to commit crimes. Although a large number of participants in the study were attracted to the monetary gains and the need to protect family members and friends, there is a tipping point. In reaching a state of readiness to violate a law, individuals will need to evaluate whether an offense will be a solution to their needs. In other words:

It can therefore be predicted that if the expected utility of illegal actions exceeds that of the legal alternatives, an individual will be more likely to decide to engage in a specific crime at a later date (i.e., they will have reached a state of “readiness”)[41].

Information security research needs a major and fundamental shift toward a reconceptualization of deterrence to account for rational forces and restrictive deterrence [41]. One interesting area for research is how potential opportunities to engage in internal computer abuse are shaped by technical skills and the jobs of the insiders. It is also worth considering whether these same employees with the passage of time have been able to contemplate faults in the systems. People in jobs for a long time understand the deficiencies in all aspects of a system, including security flaws. Job movement is one way to deal with this issue, but in the interest of specialization and productivity, moving people around is rarely embraced as a mechanism to increase security.

## Discussion

### Principal Findings

This study aimed to examine the role that monetary incentives play in violating HIPAA regulations and privacy laws in the next generation of employees. Scenarios were developed for 5 situations to determine whether monetary incentives could influence subjects to obtain health care information and to release that information. Approximately 35% to 46% of the 523 survey participants indicated that there is a price, ranging from US $1000 to over US $10 million, that is acceptable for violating HIPAA laws. In addition, subjects were also asked about their perceived probability of getting caught for violating HIPAA laws. More than 50% of the participants indicated that the probability of getting caught was more than 75%. Nevertheless, many of them could still be incentivized to violate HIPAA laws. The correlations between the probability of being apprehended and the level of the monetary incentive required for violating HIPAA ranged from 0.14 to 0.43.

In the pilot study consisting of 64 medical residents and 32 executive MBA candidates, just 6% (6/96) of the participants would succumb to monetary incentives and violate HIPAA laws. The amount of money required to incentivize medical residents and executives would also be large, ranging from US $50,000 to US $1 billion.

Between 25% and 30% of the subjects in the main study could be incentivized to violate HIPAA laws if they were offered over US $100,000. This is a substantial amount of money, and it is unlikely that such a sum would be offered to trusted insiders to violate privacy laws. The bad news is that although the number of HIPAA privacy breaches detected is declining, the dollar values of losses are escalating.

In general, individuals who perceive that there is a high probability of being caught are less likely to release private information. The implication is that technology and improvements in organizational processes could increase the perception of the probability of getting caught. The bad news is that approximately 15% of the subjects in the study would receive money, even if there is a 93% or greater chance of being caught.

Moreover, computer knowledge is not necessary because of the availability of *crime as a service*. Third-party providers can be used in cyberattacks [64]. Anyone can hack and attack and become an amateur hacker using simple automated programming tools and distributed denial-of-service–for-hire attacks and by obtaining billions of compromised passwords from the dark web [65]. Trusted insiders could provide the needed entrée for third-party providers of cyberattacks.

Our last finding is that there is a small chance of being caught, and there is an even smaller chance of being convicted. One security expert estimates that for every individual who gets caught, 10,000 people go free and that for every 1 individual who is successfully prosecuted, 100 get off scot-free or just receive a warning [66].

Between April 2003 and July 2018, there were 186,453 health information privacy complaints submitted to the US Department of Health and Human Services [67]. Of these complaints, 37,670 were investigated, resulting in 26,152 (69%) corrective actions. The Office of Civil Rights has imposed civil penalties of US $78,829,182 for just 55 cases. During that same period, the Department of Justice received 688 cases from the Office of Civil Rights for further criminal investigation. It is very difficult to obtain details about the disposition of criminal HIPAA violations. We conducted a search at the Department of Justice [68] using *HIPAA* as a keyword on their website where the Department of Justice has obtained fines and jail time. As illustrated in Multimedia Appendix 3, there were only 11 cases with fines and jail time.

Most of the subjects in our study thought that there was a high probability of being caught for violating HIPAA laws. For example, in the nursing scenario, 30% (157/523) of the participants indicated that there was a 93% or higher chance of getting caught. Clearly, this is not the case. People, even experts, consistently misestimate statistical probabilities, even when there is new contrary evidence.

### There Is Often a Price

Our results suggest that many people have a price. It may be a significant amount of money, or it may be a situation where a family member or friend needs critical medical assistance. Monitoring credit reports is a very invasive and controversial practice, but some companies are turning to credit monitoring as a way to counter breaches prompted by financial gain, although several states have taken steps to ban or limit employer access to credit reports.

The results suggest that the subjects in this sample responded rationally to the mother and the best friend scenarios. They just discounted the negative consequences of getting caught, and they attached a very high value to the lives of their mother and best friend. They also acted rationally in the first 3 scenarios. Some people indicate that there was a low probability of getting caught, but many of those people would still not participate in illegal activities. This result may be related to the Black Swan phenomenon [69]. There may be a low probability of getting caught, but the impact of getting caught could have serious long-term consequences and might be perceived, as such, by some individuals. Fines, possible prison time, loss of a job, and difficulty securing a job in the future can result in high monetary costs and social isolation.

Although there are mechanisms for reporting violations, this is still a complex problem. Organizations need to use educational campaigns as well as monitoring and enforcement strategies that strike the proper balance of protecting health care information and protecting the privacy of individuals against inadvertent violation of HIPAA laws.

Our results illustrate the importance of providing both preventive and deterrent information to increase HIPAA compliance [70]. The key will be to implement organizational procedures and constantly monitor and develop educational and training programs that will provide the appropriate frequency and intensity of deterrent information so that employees will not ignore but will embrace HIPAA compliance.

### The Challenge Ahead

The protection of personal information is a significant challenge because this information is ubiquitous, and that information has a monetary value. Businesses use this information to target customer segments. Nonprofits use this information to increase the effectiveness of fundraising campaigns. The dark side of the abundance of personal information is that this information can be compromised and retrieved by insiders and external hackers. Insider threats can come from outside infiltrators who become insiders by phishing and social networking attacks. However, they can also come from insider threats, resulting from homegrown malicious employees who intentionally want to compromise a system for profit and for a variety of reasons, including hacktivism and thrill motives. In many instances, breaches occur because of negligence, for example, some people do not know that they are not supposed to maintain social security numbers in a temporary file or email a medical diagnosis to another doctor without obtaining permission.

Our results suggest that there is a high probability that compromises can occur when employees are presented with monetary incentives, given the right context. These results have serious implications because many security breaches are from insiders [42]. Given that the greatest challenge to organizations is insider threats, the results of this study are provocative.

There are some steps that organizations can take to reduce the chance of security breaches. They can use both preventive and deterrent controls to reduce the probability of minor and major events [71]. Preventive controls impede criminal behavior by forcing the perpetrator to deplete resources [17]. Organizations must have preventive controls in place. These preventive controls include sophisticated monitoring systems technologies and constant attention to authentication protocols to prevent unauthorized access to buildings, software, and databases. Organizations usually focus on preventives because preventives can be implemented, and they are under the control of the organization. This is in contrast to deterrent strategies that focus on the apprehension and punishment of perpetrators as well as on education, legal campaigns, and fear appeals. Developing security education, training, and awareness is always a challenge. The key is to focus continually on health information security awareness [70]. It is not enough to have employees complete a web-based or even an in-person security training class. Employees need to be immersed in security training, receive feedback, and interact socially with other employees on security issues if the training is to be successful [72]. Some organizations are taking very aggressive steps to counter insider threats from malicious employees, negligent users, and infiltrators. They install software that tracks user logins, monitors file and database usage locally and in the cloud, records web activity, and regularly monitors email activity. These systems, in addition to recording activity, can also be used to send out alerts involving unusual behavior by insiders.

## Appendix

Multimedia Appendix 1 Treatment.

Multimedia Appendix 2 Example of a web-based questionnaire for the nurse aide scenario.

Multimedia Appendix 3 Criminal penalties levied by the Department of Justice.

## Abbreviations

HIPAA: Health Insurance Portability and Accountability Act
