# Correction: Good News and Bad News About Incentives to Violate the Health Insurance Portability and Accountability Act (HIPAA): Scenario-Based Questionnaire Study

**DOI:** 10.2196/24243

**Published:** 2020-09-15

**Authors:** Joana Gaia, Xunyi Wang, Chul Woo Yoo, G Lawrence Sanders

**Affiliations:** 1 State University of New York at Buffalo Buffalo, NY United States; 2 Hankamer School of Business Baylor University Waco, TX United States; 3 Florida Atlantic University Boca Raton, FL United States

In “Good News and Bad News About Incentives to Violate the Health Insurance Portability and Accountability Act (HIPAA): Scenario-Based Questionnaire Study” (JMIR Med Inform 2020;8(7):e15880) the authors noted two errors.

In the original article, the incorrect state was listed for the affiliation of author Chul Woo Yoo. The original affiliation was:

Florida Atlantic University, Boca Raton, NY, United States

The corrected affiliation is:

Florida Atlantic University, Boca Raton, FL, United States

As well, the scenario descriptions in Textbox 1 were not complete. The original descriptions for the scenarios were:

Scenario 1: Nurse’s aide, no personal context

Suppose you are a nurse’s aide at a hospital, and you earn US $30,000 per year. A friend asks you to get them some information on a patient you have been caring for. What amount of money would you receive to make this acceptable?

Scenario 2: Doctor, no personal context

Suppose you are a doctor at a hospital, and you earn US $200,000 per year. A very close friend asks you to access patient information to help them in an upcoming legal battle. What amount of money would you receive to make this acceptable?

The complete descriptions for the scenarios are:

**Scenario 1: Nurse’s aide, no personal context**

Suppose you are a nurse’s aide at a hospital and you earn US $30,000 per year. A friend asks you to get them some information on a patient you have been caring for. What amount of money would you receive to make this acceptable?

**Scenario 2: Doctor, no personal context**

Suppose you are a doctor at a hospital and you earn US $200,000 per year. A very close friend asks you to access patient information to help them in an upcoming legal battle. What amount of money would you receive to make this acceptable?

**Scenario 3: Insurance local celebrity, no personal context**

Suppose you work for an insurance company and make US $60,000 per year. A relative asks you to get insurance data on a famous local celebrity from the organization you work for. What amount of money would you receive to make this acceptable?

**Scenario 4: Your mother needs an experimental treatment, personal context**

Your mother has just been diagnosed with a rare condition that causes kidney failure and is fatal if untreated. This condition can be treated, but the treatment is still considered experimental and is therefore not covered by health insurance, nor is it eligible for any type of financial assistance. The treatment is available both nationally and internationally and costs US $100,000. A media outlet approaches you to get information about a famous politician and offers to pay you US $100,000 for that information. This money can save your mother’s life. Would you accept the payment from the media outlet and give the money to your mother?

**Scenario 5: Best friend needs air medical transportation, personal context**

Your best friend has been in an all-terrain vehicle accident in a rural area of Kansas. He or she has life-threatening injuries and needs air medical transportation to receive lifesaving medical care. The medical air evacuation is not covered by insurance and costs US $50,000. Your best friend will not survive ground transportation or local medical care. A media outlet offers you US $50,000 to obtain the health care records of a famous reality television star. This money can save your best friend’s life. Would you accept the payment from the news outlet to give the money to your best friend?

**Each scenario also included the following question:**

What do you think is the likelihood of getting caught if you accept the money?

Extremely unlikely (0%)

Moderately unlikely (7%)

Slightly unlikely (25%)

Neither likely nor unlikely (50%)

Slightly likely (75%)

Moderately likely (93%)

Extremely likely (100%)

The correction will appear in the online version of the paper on the JMIR Publications website on September 15, 2020, together with the publication of this correction notice. Because this was made after submission to PubMed, PubMed Central, and other full-text repositories, the corrected article has also been resubmitted to those repositories.

