# Supplementary figures and images for: Good News and Bad News About Incentives to Violate the Health Insurance Portability and Accountability Act (HIPAA): Scenario-Based Questionnaire Study
Source: JMIR Med Inform. 2020 Jul 20;8(7):e15880. doi: 10.2196/15880 (PMC7399953; doi:10.2196/15880)

Appendix 1: Treatment


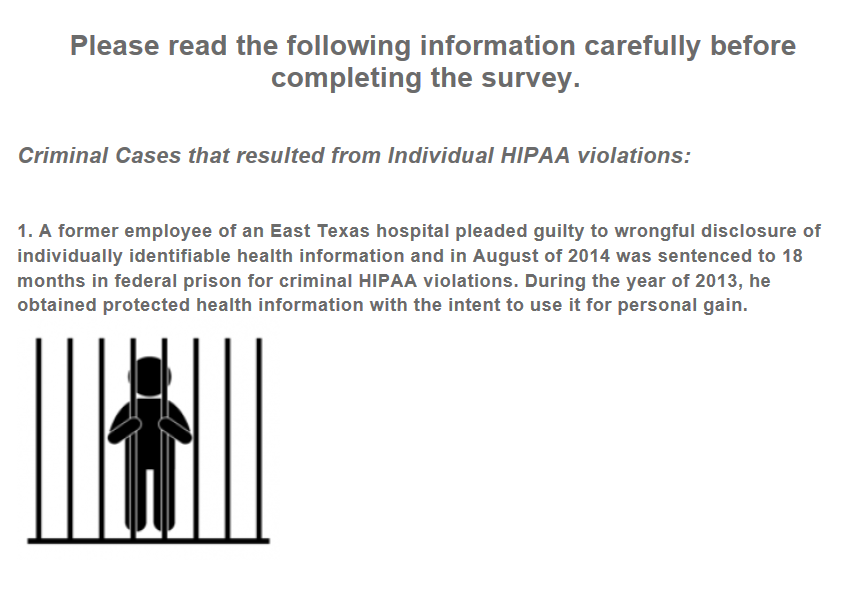


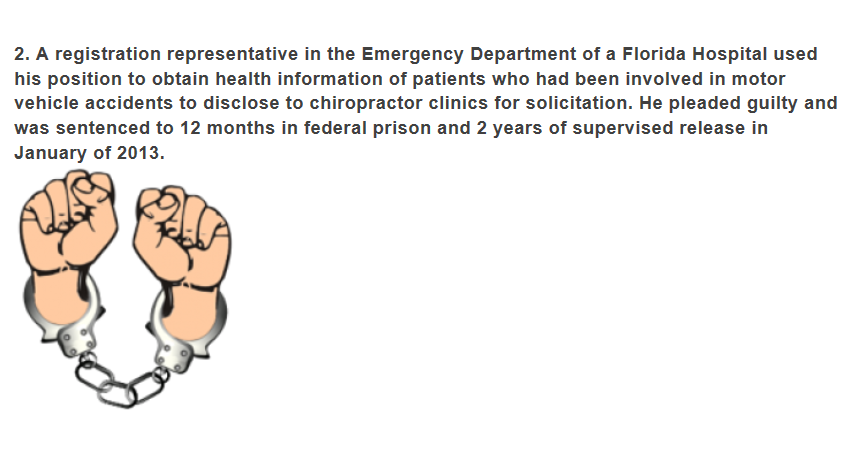


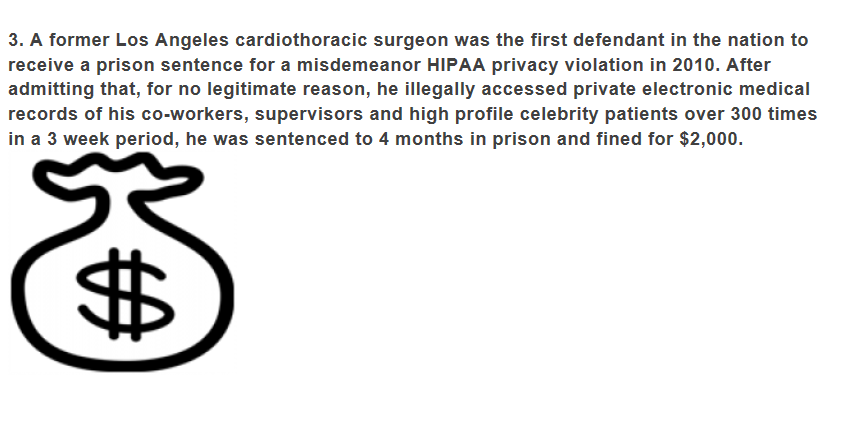

Supplement: Multimedia Appendix 1 [file medinform_v8i7e15880_app1.docx]

Appendix 2: Example of online questionnaire for Nurses Aid scenario


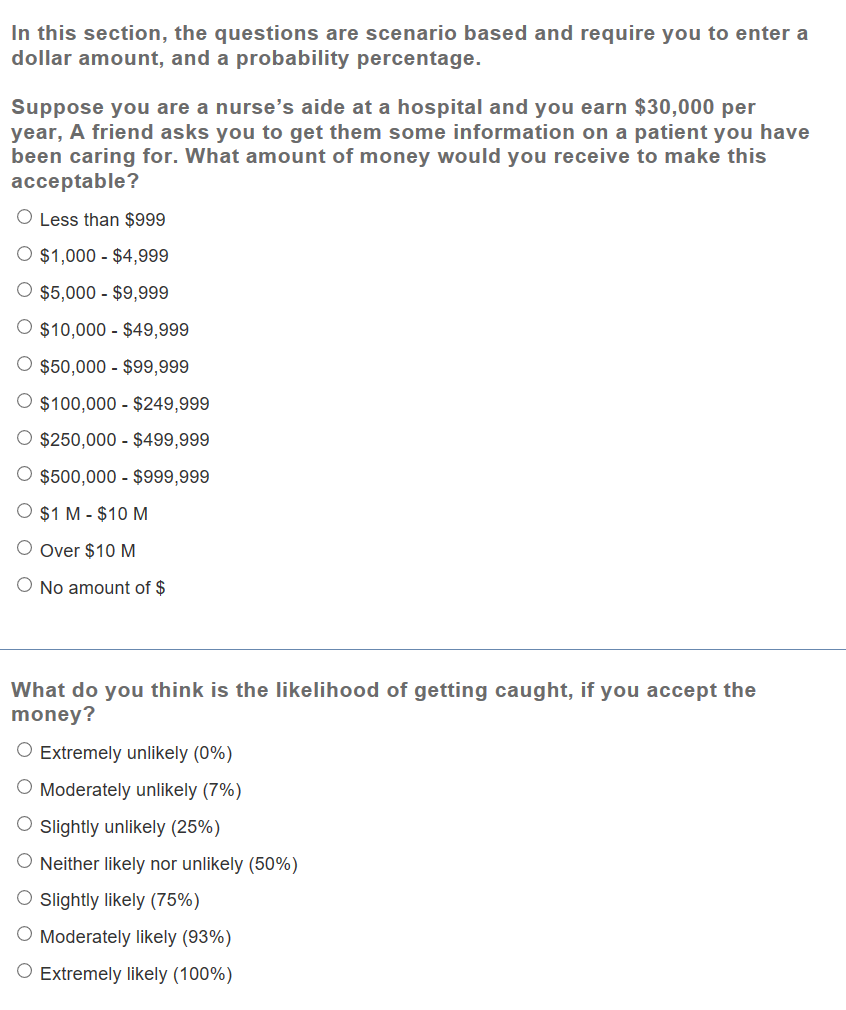

Supplement: Multimedia Appendix 2 [file medinform_v8i7e15880_app2.docx]
