# Supplementary material for: Good News and Bad News About Incentives to Violate the Health Insurance Portability and Accountability Act (HIPAA): Scenario-Based Questionnaire Study
Source: JMIR Med Inform. 2020 Jul 20;8(7):e15880. doi: 10.2196/15880 (PMC7399953; doi:10.2196/15880)
Supplement: Multimedia Appendix 3 [file medinform_v8i7e15880_app3.docx]

Appendix 3: Criminal Penalties levied at Department of Justice

| **No.** | **Year** | **Job description** | **Benefit** | **Jail Time** | **Fine or Forfeit** |
| --- | --- | --- | --- | --- | --- |
| 1 | 2012 - 2013 | Hospital Employee | Personal gain | 18 months | N/A |
| 2 | 2011 | Pharma company manager | Personal gain of $60,000 | 1 year | Fine $10,000 |
| 3 | March 2013 | Hospital Financial counselor | Exchange for drug proceeds | 2 years | N/A |
| 4 | 2011 | Pharma company district manager | Personal gain of $60,000 | 8 months of home confinement | Forfeit $21,500 |
| 5 | 2011 | Pharma company district manager | Personal gain of $100,000 and was promoted to senior district manager | 8 months of home confinement | Forfeit $28,237; fine $10,000 |
| 6 | 2003-2007 | Medical equipment company owner and manager | Personal gains (multi-million-dollar home, a half-million-dollar pension account, and personal items such as luxury cars and designer handbags) | 12 years | Forfeit $1.3 million |
| 7 | 2012 | Scheduler of hospital who had access to personal identification information | In exchange for the promise of future payments | 18 months in prison to be followed by three years of supervised release | $15,795 |
| 8 | 2012 | N/A | Personal gain | 40 months in prison followed by three years of supervised release | $174,130 |
| 9 | 2009-2013 | Pharmaceutical company owner | Personal gain | N/A | Fine of $20,742,054, forfeiture of $2 million, and $197,946 in restitution to two insurance companies. |
| 10 | 2012-2015 | Sales representative of healthcare company | In exchange for ordering or prescribing the products he promoted | 3 years | Fine of $15,000 |
| 11 | Prior to 2012 | A couple who worked as registration specialists of healthcare companies | Personal gain | 30 months | $297,957.89 in restitution to Medicare |
